# A simplified and easy‐to‐use HIP HOP assay provides insights into chalcone antifungal mechanisms of action

**DOI:** 10.1002/1873-3468.14483

**Published:** 2022-09-09

**Authors:** Thomas A. K. Prescott, Laura Anaissi‐Afonso, Keith R. Fox, Anthony Maxwell, Barry Panaretou, Félix Machín

**Affiliations:** ^1^ Royal Botanic Gardens, Kew Richmond UK; ^2^ Unidad de Investigación Hospital Universitario Ntra Sra de Candelaria Santa Cruz de Tenerife Spain; ^3^ Instituto de Tecnologías Biomédicas Universidad de la Laguna Tenerife Spain; ^4^ School of Biological Sciences University of Southampton UK; ^5^ Department of Biochemistry and Metabolism John Innes Centre Norwich UK; ^6^ School of Cancer and Pharmaceutical Sciences King's College London UK; ^7^ Facultad de Ciencias de la Salud Universidad Fernando Pessoa Canarias Las Palmas de Gran Canaria Spain

**Keywords:** 4′‐hydroxychalcone, mechanism of action, *trans*‐chalcone, transcription, yeast array

## Abstract

Elucidating the mechanism of action of an antifungal or cytotoxic compound is a time‐consuming process. Yeast chemogenomic profiling provides a compelling solution to the problem but is experimentally complex. Here, we demonstrate the use of a highly simplified yeast chemical genetic assay comprising just 89 yeast deletion strains, each diagnostic for a specific mechanism of action. We use the assay to investigate the mechanism of action of two antifungal chalcone compounds, *trans*‐chalcone and 4′‐hydroxychalcone, and narrow down the mechanism to transcriptional stress. Crucially, the assay eliminates mechanisms of action such as topoisomerase I inhibition and membrane disruption that have been suggested for related chalcone compounds. We propose this simplified assay as a useful tool to rapidly identify common off‐target mechanisms.

## Graphical abstract

Here, we demonstrate a simplified HIP HOP assay comprising 89 yeast deletion strains, each diagnostic for common cell inhibitory mechanisms of action. We test two chalcone compounds with unknown mechanisms, *trans*‐chalcone and 4′‐hydroxychalcone and show that both act through transcriptional stress. The assay is both easy and cheap to use and can help identify and eliminate common mechanisms of action.

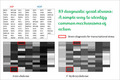

## Abbreviations

**DCF**, 2′,7′‐dichlorofluorescein

**HIP HOP**, haploinsufficiency profiling homozygous profiling

**ROI**, regions of interest

**ROS**, reactive oxygen species

**YPD**, yeast peptone dextrose

Yeast chemogenomic profiling is a powerful tool to understand the mechanism of action of both antifungal compounds and compounds exerting an inhibitory effect on human cells [1]. The budding yeast *Saccharomyces cerevisiae* is a simple and genetically tractable eukaryotic organism that is able to act as a model for conserved eukaryotic cellular biochemistry in both fungi and human cells. In a conventional yeast chemical genetic screen, genome‐wide panels of yeast deletion strains, each deleted for a different gene, are exposed to a sub‐lethal inhibitory dose of a test compound [1, 2]. The relative growth of each yeast deletion strain is then determined with the specific pattern of inhibition across all ~ 6000 yeast gene deletion strains being characteristic for the mechanism of action of the test compound [3]. For example, when a genome‐wide yeast deletion library is exposed to a sublethal dose of the microtubule targeting compound benomyl, yeast strains genetically knocked‐down for tubulin exhibit a hypersensitive phenotype and are markedly slow growing compared to other yeast deletion strains in the library [4]. The combined effects of genetic knock down of tubulin, and chemical inhibition of tubulin assembly, combine synergistically to ensure that the yeast strain carrying a gene deletion for the target (tubulin) is disproportionately slow‐growing relative to other yeast deletion strains included in the library [4]. This so called ‘chemical genetic interaction’ is an effect that has been exploited in numerous yeast chemical genetic assays to determine the mechanism of action of novel chemical inhibitors and to confirm the previously established mechanisms of action of known small molecule inhibitors [1].

Yeast chemogenomic assays such as these make use of diploid yeast possessing two copies of each gene. As both copies of an essential gene cannot be deleted, to achieve a partial knock down, heterozygous deletion strains are used in which one copy of the essential gene is deleted resulting in a 50% reduction of the protein in question. Screening with these heterozygous deletion strains is referred to as HIP (heterozygous profiling) [5]. Conversely, non‐essential genes that are not a direct target of the test compound but that act to modulate its effects, can have both copies of their gene deleted providing a complete knockdown. For example, yeast strains lacking both copies of DNA repair genes exhibit hypersensitivity to DNA damaging compounds [4]. Screening these non‐essential gene deletion strains is referred to as HOP (homozygous profiling) and can provide insights into pathways that buffer the effects resulting from inhibition of the target protein [1]. Irrespective of the configuration used, such genome‐wide screens, although powerful, are difficult to carry out in laboratories that lack specialised equipment. For example, the entire collection of ~ 6000 yeast deletion strains can be arrayed onto agar plates in the presence of a test compound but this requires specialised yeast arraying robots to achieve a high‐density array. If lower density arrays are used then larger amounts of test compound are required, which is a limitation in the natural products field where isolation of large quantities of a novel metabolite may not be possible. An alternative is to run the assay as a pooled experiment in which the unique barcodes inserted into each yeast deletion strain are used to distinguish the growth of each strain [4]. This approach has been used successfully for high‐throughput screening of compound libraries but requires relatively expensive procedures to determine the relative growth rate of each deletion strain from its barcode, and for this reason, the pooled assay format has not been widely implemented.

A potential route to simplification of chemogenomic profiling arises from the observation that not all gene deletion strains are diagnostic for a specific mechanism of action. Indeed, work carried out at the Hoepner lab at Novartis showed that a large proportion of yeast gene deletion strains respond to multiple drugs with unrelated mechanisms of action and therefore are not diagnostic [4]. Conversely, certain yeast gene deletion strains were found to be diagnostic for specific mechanisms of action and could therefore be considered ‘signature strains’ [4]. Mechanisms of action that are associated with diagnostic signature strains include highly specific ligand–protein interaction mechanisms but also include mechanisms that lack a specific protein target which commonly go unnoticed in drug screening but are important causes of ‘off‐target’ toxicity [4]. An example of this is the yeast deletion strains defective for the iron import channels which are hypersensitive to iron‐chelating drugs [6]. Iron‐chelating compounds do not target a specific protein receptor but can be an important source of off‐target toxicity, which needs to be identified early during the drug discovery process. An assay that could rapidly confirm or eliminate these types of off‐target mechanisms along with other more common mechanisms of action, and to do so with minimal workflow and cost, would be a substantial benefit to both antifungal and human cytotoxic mechanism of action elucidation. This is particularly pertinent right now due to recent evidence suggesting off‐target effects are contributing to a reproducibility crisis in mechanism of action research [7, 8].

In previous work, we showed that a minimal set of ‘signature strains’ can provide useful insights into compound mechanism of action [9]. In this study, we make use of data from large scale, genome‐wide, yeast chemical genetic screening by the Hoepfner laboratory at Novartis to provide a simplified assay comprising 89 yeast deletion strains diagnostic for common mechanisms of action including non‐protein target type mechanisms. We validate the assay using two chalcone compounds, *trans*‐chalcone and 4′‐hydroxychalcone. Both compounds exhibit antifungal activity with *Candida albicans* [10, 11] and *trans*‐chalcone is also inhibitory towards the fungal pathogen *Trichophyton rubrum* [12]. A variety of different mechanisms have been proposed for the antifungal mechanisms of chalcones. A study that examined the effect of *trans*‐chalcone on the fungal pathogen *T. rubrum* revealed changes in fatty acid synthesis that were inferred to interfere with cell membrane and cell wall integrity [12]. In another study, a methoxylated chalcone active against *Candida tropicalis* was investigated for its mechanism of action, revealing membrane damage to be the likely mechanism of action [13]. Antifungal structure activity relationship investigations of large sets of chalcones have found electron withdrawing substitutions in their B‐ring to be important for activity as well as the ability of compounds to interact with sulfhydryl groups [11, 13].

Both *trans*‐chalcone and 4′‐hydroxychalcone also have inhibitory effects in cancer cell lines, and a variety of different mechanisms have been proposed [14, 15]. Considering the diverse range of eukaryotic organisms that are susceptible to chalcones (thereby implying a conserved mechanism) those cancer cell inhibitory mechanisms associated with conserved, fundamental biochemistry would seem to be the most relevant. For chalcones such mechanisms include inhibition of sirtuins, histone deacetylases, tubulin polymerisation, CDC25, topoisomerase II (Top2), mTOR as well as DNA intercalation [16, 17, 18]. The diversity of mechanisms reported suggests a degree of promiscuity in chalcone cell inhibitory effects and emphasises the importance of assays that include non‐protein receptor type mechanisms that may be responsible for pleiotropic responses.

## Materials and methods

### Compounds

4′‐Hydroxychalcone was obtained from the internal compound collection of RBG, Kew. Purity and structure were verified using LC–MS and NMR. *trans*‐Chalcone was purchased from Sigma‐Aldrich, Gillingham, UK.

### Selection of strains for yeast array

Diagnostic yeast deletion strains were selected through a systematic examination of the web portal for: High‐resolution chemical dissection of a model eukaryote reveals targets, pathways and gene functions (see http://hiphop.fmi.ch) [4]. The web portal provides complete genome‐wide HIP HOP profiles for a diverse set of control compounds with known mechanisms of action. For each compound, strains were selected from both the HIP and HOP profile that were most sensitive to the compound in question as well as having the lowest *Z* factor, so as to provide the best diagnostic value. Where the profile presented multiple strains with high sensitivity and low *Z* factor scores, a preference was made for genes whose gene ontology term was associated with the known drug target of the compound. Where possible, selection of diagnostic yeast strains for a given mechanism of action was based on HIP HOP profiles from at least two different compounds sharing the same mechanism of action. All strains were obtained from Euroscarf, Oberusel, Germany.

### Yeast growth inhibition dose–response inhibition

Serial dilutions of the test compound in 25 μL of synthetic complete culture medium were transferred to a transparent sterile 384 well plate. Overnight cultures of yeast strains were diluted to an OD_600_ of 0.1 and 25 μL were added to each well so as to achieve a final OD_600_ in each well of 0.05. The plate was then incubated at 30 °C in a Tecan Infinite M200 plate reader (Tecan, Mannedorf, Switzerland), and the OD_600_ values recorded in nine separate locations in each well every 20 min for 22 h, with the readings at 15 h used to calculate growth inhibition relative to no treatment controls using graphpad prism software (GraphPad Prism, San Diego, CA, USA).

### Screening the yeast array

The yeast deletion strains were stored in 15% glycerol in a 96 well plate at −80 °C. The strains were arrayed onto an Omni tray containing yeast peptone dextrose (YPD) agar and incubated for 24 h at 30 °C to aid recovery and full growth. In all cases, arraying and transfer of strains from plate to plate was carried out using a 96 floating pin tool (VP Scientific, San Diego, CA, USA). The yeast array was then transferred from YPD agar onto four different Omni trays containing synthetic complete agar medium, and after a further 24 h growth at 30 °C these arrays were used to inoculate four separate 96 well plates each containing 100 μL of liquid synthetic complete medium per well, which were each incubated for 24 h at 30 °C. Finally, four new 96 well plates containing each test compound in 95 μL of synthetic complete media per well were inoculated with 5 μL of diluted yeast culture so as to achieve a starting cell density in each well corresponding to an OD_600_ value of 0.05 and a final concentration of each test compound equal to its respective IC_50_ (153.5 μm for *trans*‐chalcone and 97 μm for 4′‐hydroxychalcone). The four replicate plates corresponding to each test compound and four no compound controls plates were incubated for 14 h at 30 °C. DMSO concentration was normalised between all plates. After 14 h, the OD_600_ value in each well was read in nine separate locations using a Tecan Infinite M200 plate reader. Inhibition for each yeast deletion strain was then calculated relative to no compound DMSO controls.

### Propidium iodide membrane damage assay

Serial dilutions of test compounds and DMSO solvent controls were prepared in a black 96 well plate at 2× final concentration in 125 μL. A culture of BY4743 [Mata/α *his3Δ1/his3Δ1; leu2Δ0/leu2Δ0; met15Δ/MET15 LYS2/lys2Δ0; ura3Δ0/ura3Δ0* (Euroscarf)] yeast cells suspended in Dulbecco's phosphate buffered saline at a concentration of OD_600_ 3.0 was prepared and 125 μL cell suspension added to plate along with propidium iodide to achieve a final concentration of 4 μg·mL^−1^ propidium iodide. Fluorescence was followed at Ex 535 and Em 617 with reads every 10 min for 6 h at 30 °C using a Tecan Infinite M200 plate reader.

### DNA intercalation and melting curve assays

Compounds were assessed for their ability to intercalate into DNA using a wheatgerm DNA topoisomerase I unwinding assay (Inspiralis, Norwich, UK). Relaxed and supercoiled forms of plasmid pBR322 (0.5 μg) were incubated with 2.5 units wheatgerm topoisomerase I in 50 mm of Tris–HCl (pH 7.9), 50 mm of NaCl, 1 mm of EDTA, 1 mm of DTT and 20% (v/v) glycerol, in a total volume of 30 μL at 37 °C for 30 min, in the presence of 0, 1, 10, 50 μm compound. Each reaction was then extracted by the addition of 20 μL of water and 50 μL of butanol to remove the compound, then stopped with the addition of 30 μL chloroform/isoamyl alcohol (24 : 1) and 30 μL Stop Dye [40% sucrose, 100 mm Tris–HCl (pH 7.5), 10 mm of EDTA, 0.5 μg·mL^−1^ of bromophenol blue], before being loaded onto a 1% agarose gel in TAE (40 mm of Tris base, 20 mm of glacial acetic acid, 1 mm of EDTA) and run at 90 V for 90 min. Gels were then stained in 1 μg·mL^−1^ of EtBr in H_2_O for 20 min and de‐stained in TAE for 5 min, before imaging with a UV gel documentation system (Syngene, Cambridge, UK).

Fluorescence melting curves were performed as described previously [19]. The oligonucleotide duplex (0.2 μm) was annealed in 10 mm of phosphate buffer pH 7.4 containing 200 mm of NaCl in the presence of different chalcone concentrations in a total volume of 20 μL. Samples were heated in a Roche LightCycler (Roche Diagnostics Ltd, Burgess Hill, UK) at 0.1 °C·s^−1^ and the fluorescence recorded.

### Yeast topoisomerase II assay

Compounds were assessed for their ability to inhibit yeast DNA topoisomerase II using a decatenation and a relaxation assay (Inspiralis). One unit of yeast topoisomerase II was incubated with 0.2 μg of kDNA (kinetoplast) in a 30 μL reaction at 30 °C for 30 min under the following conditions: 20 mm of Tris–HCl (pH 7.9), 200 mm of KCl, 10 mm of MgCl_2_, 4% (v/v) glycerol, 1 mm of ATP. Compounds were added to the reaction before the addition of enzyme. Reactions were terminated and samples analysed as described above. Relaxation reactions were carried out as described for the decatenation assay except that the substrate was 0.5 μg supercoiled pBR322 DNA.

### Rad52 hypersensitivity assay

Two isogenic strains were used for testing Top2 poisoning: The reference *TOP2 RAD52* wild‐type strain and *TOP2 rad52*Δ. All strains carry the *ISE2* mutation, which affects pleiotropic resistance and confers an elevated sensitivity to etoposide [20]. Strains were pre‐grown in YPD and diluted to 0.05 OD_600_ in 96 well format to begin recording real‐time growth curves in a TECAN Spark multiplate reader. Final concentration of chalcones was 100 μm, and etoposide concentration was titrated to be equipotent to the chalcones in the wild‐type *ISE2* strain. The equipotent etoposide concentration was established to be 300 μm by both maximum growth rate and area under the growth curve. In all cases, we used stocks of drugs resuspended in DMSO and the final DMSO concentration in the plate wells was 1% v/v. The temperature of the assays was 30 °C and the growth was recorded for 24 h. Growth rate was estimated as the maximum slope obtained after applying linear regression on sets of five consecutive OD_600_ measurements. Statistical significance was calculated with the two‐sided unpaired *t* test.

### Oxidative stress assay

Briefly, wild‐type BY4741 (*Mata his3Δ1; leu2Δ0; met15Δ; ura3Δ0* [Euroscarf]) and *yap1*Δ strains were plated as a lawn (~ 50 cells·mm^−2^) on either YPD or YPGly 90 mm Petri dishes. Compounds were spotted at the indicated amounts on the positions defined in the figures. For 10 nmol, 1 μL from 10 mm stocks in DMSO was spotted. For 20, 40 and 80 nmols, 1, 2 and 4 μL from 20 mm stocks were spotted, respectively. Corresponding volumes of DMSO were spotted as controls. Plates were incubated at 30 °C under either normoxia or anoxia conditions. Anoxia was achieved in a sealed bag using the AnaerocultTM A mini kit (Merck, Darmstadt, Germany). YPD plates under normoxia were incubated for 3 days, taking pictures every 24 h. YPD plates under anoxia were incubated for 3 days before breaking the bag and taking a single picture. YPGly plates were incubated for 6 days, taking pictures every 72 h.

To quantify inhibition, we used fiji/imagej (bethseda, md, usa) to measure intensity of darker areas around the spots. In this way, we overcame the subjective assessment of total versus partial inhibition halos. A straight line was drawn across the halos to obtain the pixel intensity profile and calculate the area of the profile darker than the lawn. We then subtracted either DMSO spot controls from the corresponding compounds and plotted the resulting profile area.

### Determination of reactive oxygen species

The assay used DCFH‐DA which is a reduced, acetylated and permeable non‐fluorescent form of 2′,7′‐dichlorofluorescein (DCF). Upon entry into the cell, DCFH‐DA is deacetylated and retained; if reactive oxygen species (ROS) is present it gets oxidised as well, yielding the green fluorescent DCF.

An asynchronous culture of wild‐type cells was treated with the corresponding drugs in the presence of 10 μg·mL^−1^ DCFH‐DA and 3 μg·mL^−1^ PI propidium iodide which labels dead cells in red. Two hours later, cells were harvested, washed once with phosphate buffered saline (PBS 1×) and visualised directly on a Leica DMI6000 fluorescence microscope (Leica Microsystems GmbH, Wetzlar, Germany) with narrow bandpass filters for DCF and PI.

To quantify ROS, regions of interest (ROIs) were manually drawn around cells in the bright field. ROIs that were negatively stained with PI (> 95% cells in all cases) were then used to quantify mean DCF intensities. Finally, DCF intensities were normalised to the mean DMSO intensity and box plotted, with each data point representing an individual cell (~ 100 cells counted for each condition). In the box plot, centre lines show the medians; box limits indicate the 25th and 75th percentiles; whiskers extend to minimum and maximum values; and notches represent the 95% confidence interval for each median. Non‐overlapping notches give roughly 95% confidence that two medians differ. The box plot was generated at http://shiny.chemgrid.org/boxplotr/

### Cell‐free translation assay

The cell‐free translation assay was carried out using the Rabbit Reticulocyte Lysate protein expression system (Promega, Southampton, UK), which is a nuclease‐treated and contains an energy‐regenerating system, a mixture of tRNAs, hemin, potassium chloride and magnesium acetate. The kit was adapted to a 384 well plate format by scaling down recommended kit reagent volumes to 15 μL and using an adhesive foil to prevent evaporation. The Rabbit Reticulocyte Lysate mixture was incubated with compound dilutions and DMSO solvent vehicle and luciferase control RNA at 30 °C for 90 min. The luciferase control RNA (also supplied with the kit) is an uncapped RNA containing a 30‐base poly(A) tail suitable for *in vitro* translation. After 90 min, 5 μL of the reaction mixture were transferred to a white luminescence 384 well plate containing 75 μL per well 150 μg·mL^−1^ d‐luciferin sodium salt in nuclease free water. Luciferase luminescence was then monitored using a Tecan Infinite M200 plate reader.

### GFP transcription/translation assay

GFP was placed under the control of the *GAL1* promoter, inserted into a centromeric *URA3* vector with a built‐in transcriptional terminator (YCplac33T). This recombinant was formed by using a HiFi assembly kit (New England Biolabs, Ipswich, MA, USA) to ligate the *GAL1* promoter amplified from pYES2 (Thermo Fisher Scientific, Waltham, MA, USA), codon optimised enhanced GFP (amplified from pKT127; Euroscarf) and *Bam*HI/*Pst*1 digested YCplac33T. This was transformed into BY4742 Matα *his3Δ1; leu2Δ0; lys2Δ0; ura3Δ0* (Euroscarf). Transformants were selected on YNB media supplemented with drop‐out mix minus uracil (Melford, Ipswich, UK). To perform the assay, replicate cultures of the yeast strain were grown in YNB with no uracil and 2% raffinose media to reach an OD_600_ of 3.7 and 90 μL added to wells of a black 96 well plate. The cells were then induced with 9 μL 20% w/v galactose solution with serial dilutions of drugs added in 1 μL volumes. GFP fluorescence (ex/em 483/535 nm) was then measured at in a Tecan Infinite M200 plate reader every 10 min for 4 h.

## Results

### A panel of 89 yeast deletion strains is diagnostic for inhibitory mechanisms associated with diverse areas of cell biology

Previous high‐throughput genome‐wide HIP HOP profiling carried out by Hoepfner et al. [4] at the Novartis Institutes for Biomedical Sciences has provided high‐quality chemical genetic profiles for a diverse set of compounds, including a set of compounds with known mechanisms of action. The advantage of such a large data set is that it allows the identification of both diagnostic and non‐diagnostic strains. Of the more than ~ 6000 yeast gene deletions strains present in the screening set, a large proportion respond with a hypersensitive phenotype to compounds with unrelated mechanisms of action and are therefore not diagnostic. Conversely, a much small number of strains display a hypersensitive phenotype only in response to compounds with a common mechanism of action and can therefore be considered diagnostic for that mechanism of action. Listed below in Table 1 are a set of 89 yeast deletion strains obtained from both the HIP and HOP profiles that form a simplified HIP HOP panel. Each strain is derived from the diploid parental strain BY4743 and contains either a deletion of one copy of the target gene (HIP panel), or has both copies of the target gene deleted (HOP panel). Validated control compounds are listed next to each gene deletion strain.

**Table 1:** A list of HIP HOP yeast gene deletion strains that are diagnostic for indicated mechanisms. The table lists the gene, its systematic name and Euroscarf accession number along with validated control compounds that induce hypersensitivity in the corresponding HIP or HOP strain. The strains were obtained from a systematic search of the database provided by the Hoepfner laboratory at Novartis [4].

Area of cell biology	Gene	HIP or HOP	Validated positive control compounds	Systematic name	Acc. no.
Membrane disruption	*NEO1*	HIP	Amitriptyline, chlorpromazine	*YIL048w*	Y21441
Membrane disruption	*PIK1*	HIP	Amitriptyline, benzethonium, chlorpromazine	*YNL267w*	Y26958
Membrane disruption	*TIM54*	HIP	Amitriptyline, chlorpromazine	*YJL054w*	Y21369
Membrane disruption	*CMD1*	HIP	Gefitinib	*YBR109c*	Y23248
Membrane disruption	*TDA10*	HIP	Gefitinib	*YGR205w*	Y24835
Membrane disruption	*LEM3*	HOP	Benzethonium, gefitinib	*YNL323w*	Y31121
Membrane disruption	*TRP4*	HOP	Dyclonine, fenpropimorph	*YDR354w*	Y34191
Vacuolar ATPases	*VMA4*	HIP	Leucanicidin, bafilomycin A1, concanamycin A, hygrolidin	*YOR332w*	Y21629
Vacuolar ATPases	*RAV2*	HOP	Leucanicidin, bafilomycin A1, concanamycin A, hygrolidin	*YDR202c*	Y36970
Mitochondria	*TOM40*	HIP	Ethidium bromide, niclosamide, lysolipin X	*YMR203w*	Y20789
Mitochondria	*ATP2*	HIP	Ethidium bromide	*YJR121w*	Y26924
Mitochondria	*ATP1*	HIP	Ethidium bromide	*YBL099w*	Y23125
Mitochondria	*MIA40*	HIP	Dequalinium chloride, ethidium bromide, lysolipin X	*YKL195w*	Y27033
Mitochondria	*MRPL19*	HIP	Niclosamide	*YNL185c*	Y22027
Mitochondria	*ATP1*	HOP	Strobilurin B, ethidium bromide, myxothiazol A	*YBL099w*	Y33125
Mitochondria	*ATP2*	HOP	Strobilurin B, ethidium bromide	*YJR121w*	Y36924
Mitochondria	*ATP11*	HOP	Strobilurin B, myxothiazol A	*YNL315c*	Y37319
Mitochondria	*MRPL17*	HOP	Dequalinium chloride, niclosamide	*YNL252c*	Y36465
Mitochondria	*MRPS12*	HOP	Dequalinium chloride, niclosamide	*YNR036c*	Y35411
Metal chelation	*FET3*	HOP	Deferasirox, exjade, trichostatin A, curcumin, EDTA	*YMR058w*	Y36192
Metal chelation	*MAC1*	HOP	Deferasirox, exjade, trichostatin A, 1,10‐phenanthroline monohydrate, deferiprone, EDTA	*YMR021c*	Y30596
Metal chelation	*ATF1*	HOP	Deferasirox, exjade, trichostatin A, 1,10‐phenanthroline monohydrate, deferoxamine, deferiprone, EDTA	*YGL071w*	Y34438
Metal chelation	*FTR1*	HOP	Deferasirox, exjade, trichostatin A	*YER145c*	Y36142
Metal chelation	*CCC2*	HOP	Deferasirox, exjade, trichostatin A, EDTA	*YDR270w*	Y33629
Metal chelation	*GEF1*	HOP	Deferasirox, exjade, trichostatin A, deferiprone	*YJR040w*	Y36838
Metal chelation	*CTR1*	HOP	EDTA, deferasirox, exjade	*YPR124w*	Y35539
Microtubules	*TUB2*	HIP	Epothilon B derivative	*YFL037w*	Y25658
Microtubules	*TUB3*	HIP	Epothilon B derivative, nocodazole	*YML124c*	Y26525
Microtubules	*MAD1*	HOP	Epothilon B derivative, nocodazole	*YGL086w*	Y34453
Microtubules	*PAC2*	HOP	Epothilon B derivative, nocodazole	*YER007w*	Y30329
Microtubules	*CIN2*	HOP	Epothilon B derivative, nocodazole	*YPL241c*	Y31051
Translation	*RPL19A*	HIP	Ovalicin, diazaborine, bortezomib, cycloheximide	*YBR084c‐A*	Y27156
Translation	*RPS16B*	HIP	Ovalicin, bortezomib, cycloheximide, trichothecene	*YDL083c*	Y23780
Translation	*RPL31A*	HIP	Anisomycin, bortezomib, cycloheximide, verrucarin A, borrelidin	*YDL075w*	Y23772
Translation	*IML2*	HIP	Anisomycin, bortezomib, cycloheximide, verrucarin A, trichothecene, AN2690	*YJL082w*	Y21341
Translation	*RPS24A*	HOP	Bortezomib, trichothecene	*YER074w*	Y30214
Translation	*NPL3*	HOP	Bortezomib, cycloheximide, verrucarin A, trichothecene, AN2690	*YDR432w*	Y34268
Translation	*RPS10B*	HOP	Verrucarin A, trichothecene	*YMR230w*	Y30816
Translation	*THR4*	HOP	Borrelidin, AN2690	*YCR053w*	Y37186
Translation	*SLH1*	HOP	Anisomycin, cycloheximide, trichothecene	*YGR271w*	Y37290
Fatty acid synthesis	*ERG11*	HIP	Cyproconazole, voriconazole, tubulazole, clotrimazole, fluconazole	*YHR007c*	Y26604
Fatty acid synthesis	*SET6*	HIP	Cyproconazole, voriconazole, tubulazole, clotrimazole, fenpropimorph, fluconazole	*YPL165c*	Y22087
Fatty acid synthesis	*SEC13*	HIP	Myriocin derivative	*YLR208w*	Y24157
Fatty acid synthesis	*SEC31*	HIP	Myriocin derivative	*YDL195w*	Y23893
Fatty acid synthesis	*SUR4*	HOP	Myriocin derivative, fluvastatin, lescol, fluconazole, TOFA, soraphen A, triclosan	*YLR372w*	Y35281
Fatty acid synthesis	*LRO1*	HOP	Triclosan	*YNR008w*	Y35383
Ionophores	*YOR1*	HIP	Enniatin derivative, enniantin B1, enniatin A1, antibiotic X‐206, azalomycin B	*YGR281w*	Y25933
Ionophores	*YOR1*	HOP	Enniatin derivative, enniantin B1, enniatin A1, antibiotic X‐206, azalomycin B	*YGR281w*	Y35933
Cell wall	*RHO1*	HIP	Ergokonin A, ascosteroside, echinocandin C derivative, mercury (II) chloride	*YPR165w*	Y25580
Cell wall	*FKS1*	HIP	Ergokonin A, echinocandin B derivative, ascosteroside, echinocandin C derivative, mercury (II) chloride	*YLR342w*	Y25251
Cell wall	*CHS3*	HOP	Ergokonin A, echinocandin B derivative, ascosteroside, echinocandin C derivative, mercury (II) chloride	*YBR023c*	Y33160
Cell wall	*SKT5*	HOP	Ergokonin A, echinocandin B derivative, ascosteroside, echinocandin C derivative, mercury (II) chloride	*YBL061c*	Y33087
Cell wall	*CHS6*	HOP	Ergokonin A, echinocandin B derivative, ascosteroside, echinocandin C derivative	*YJL099w*	Y31324
DNA intercalation and transcriptional stress	*REI1*	HIP	Quinacrine	*YBR267w*	Y23407
DNA intercalation and transcriptional stress	*NMD3*	HIP	Quinacrine, Hoechst 33258, cryptolepine	*YHR170w*	Y26418
DNA intercalation and transcriptional stress	*SSL2*	HIP	Ravidomycin, doxorubicin, actinomycin D	*YIL143c*	Y22302
DNA intercalation and transcriptional stress	*LSG1*	HIP	Quinacrine, cryptolepine	*YGL099w*	Y24466
DNA damage	*MMS22*	HIP	Camptothecin, zeocin, actinomycin D	*YLR320w*	Y25229
DNA damage	*RAD51*	HIP	Ravidomycin, doxorubicin, mechlorethamine, zeocin, actinomycin D	*YER095w*	Y26401
DNA damage	*RAD54*	HOP	Ravidomycin, camptothecin, aclarubicin, doxorubicin, mechlorethamine, methyl methanesulfonate, zeocin	*YGL163c*	Y34530
DNA damage	*RAD55*	HOP	Ravidomycin, camptothecin, aclarubicin, doxorubicin, mechlorethamine, methyl methanesulfonate, bleomycin A2, zeocin, actinomycin D	*YDR076w*	Y34011
DNA damage	*RAD57*	HOP	Ravidomycin, camptothecin, aclarubicin, doxorubicin, mechlorethamine, methyl methanesulfonate, bleomycin A2, zeocin, actinomycin D	*YDR004w*	Y33944
DNA damage and transcriptional stress	*SNF7*	HOP	Bleomycin A2, zeocin, actinomycin D	*YLR025w*	Y31580
DNA damage and alkylation	*MMS1*	HOP	Camptothecin, aclarubicin, doxorubicin, mechlorethamine	*YPR164w*	Y35579
DNA alkylation	*MMS2*	HOP	Mechlorethamine, methyl methanesulfonate	*YGL087c*	Y34454
Topo 1 poisoning	*TOP1*	HOP	Camptothecin (resistant)	*YOL006c*	Y31697
Chaperones	*STI1*	HIP	Radicicol, geldanamycin	*YOR027w*	Y21803
Chaperones	*STI1*	HOP	Radicicol, geldanamycin	*YOR027w*	Y31803
Kinases	*PKC1*	HIP	Staurosporin, TAN‐999	*YBL105c*	Y23133
Kinases	*SAC7*	HOP	Staurosporin, TAN‐999	*YDR389w*	Y34225
Nucleobase synthesis	*AMD1*	HIP	6‐Azauridine, 6‐azauracil	*YML035c*	Y27055
Nucleobase synthesis	*HAM1*	HOP	6‐Azauridine, 6‐azauracil	*YJR069c*	Y36893
Actin cytoskeleton	*ACT1*	HIP	Latrunculin A, chondramide, chivosazol A	*YFL039c*	Y27075
Actin cytoskeleton	*CCT2*	HIP	Latrunculin A, chondramide, chivosazol A	*YIL142w*	Y22301
Actin cytoskeleton	*CAP1*	HOP	Latrunculin A, chondramide, chivosazol A	*YKL007w*	Y34856
Actin cytoskeleton	*AIM7*	HOP	Chondramide, chivosazol A	*YDR063w*	Y33998
Actin cytoskeleton	*AIM21*	HOP	Latrunculin A, chivosazol A	*YIR003w*	Y32339
Glycolysis/starvation	*ATP14*	HIP	2‐Deoxy‐d‐glucose, methylmercury	*YLR295c*	Y25205
Glycolysis/starvation	*ATP2*	HOP	2‐Deoxy‐d‐glucose, d‐glucose (starvation), methylmercury	*YJR121w*	Y36924
Glycolysis/starvation	*MRPL33*	HOP	2‐Deoxy‐d‐glucose, d‐glucose (starvation), methylmercury	*YMR286w*	Y30872
Blocks ion channels	*EDE1*	HIP	Pramoxin, dyclonine	*YBL047c*	Y23073
Blocks ion channels	*GCN2*	HOP	Pramoxin, dyclonine	*YDR283c*	Y33642
Blocks ion channels	*GCN3*	HOP	Pramoxin, dyclonine	*YKR026c*	Y35097
Blocks ion channels	*GCN4*	HOP	Pramoxin, dyclonine	*YEL009c*	Y30249
TOR signalling	*KOG1*	HIP	Caffeine, rapamycin	*YHR186c*	Y22880
TOR signalling	*TOR1*	HOP	Caffeine, rapamycin	*YJR066w*	Y36864
ER stress	*IRE1*	HOP	Tunicamycin, E1210/gepinacin	*YHR079c*	Y31907
ER stress	*HAC1*	HOP	Tunicamycin, E1210/gepinacin	*YFL031w*	Y35650
pH stress	*RIM101*	HOP	Salinomycin, septamycin	*YHL027w*	Y30936
Oxidative stress	*YAP1* ^a^	HOP	Hydrogen peroxide, menadione^a^	*YML007w*	Y30569

^a^ A strain lacking Yap1 was included based on separate data [28].

### Validating the simplified HIP HOP panel using the known cell wall synthesis inhibitor caspofungin

**Fig. 1:**
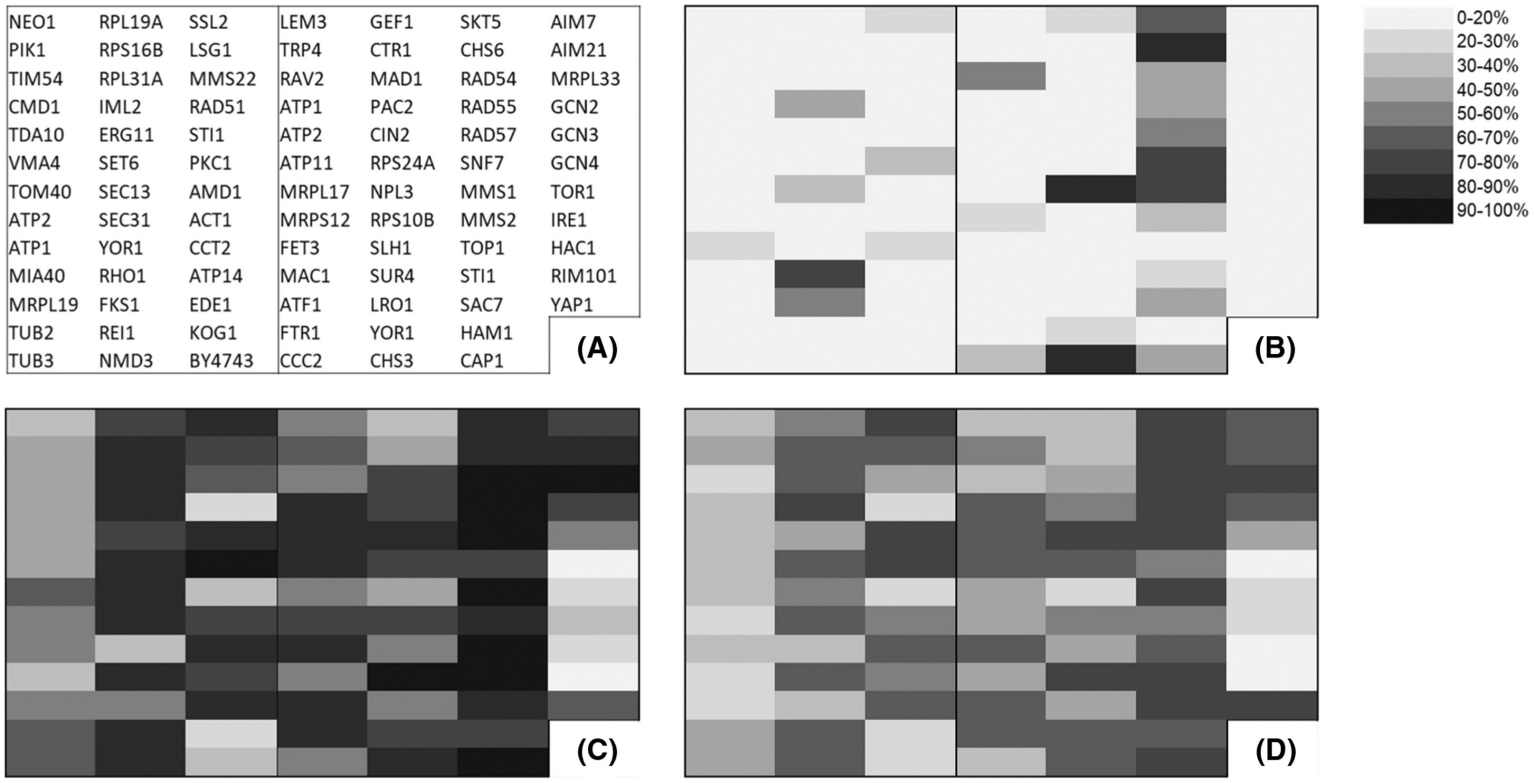
Sensitivity profiles for the chitin synthase inhibitor compound caspofungin and the two chalcone compounds each tested with the HIP HOP mini panel. Darkness or lightness of squares indicates % inhibition values obtained from four replicate screening experiments. (A) The plate map identifies the location of diagnostic gene deletion strains with heterozygous deletion strains (HIP) in left hand panel and homozygous deletion strains (HOP) in right hand panel. (B) HIP HOP sensitivity profile for caspofungin at 0.15 μm. (C) HIP HOP sensitivity profile for trans‐chalcone at the IC_50_ concentration (153.5 μm). (D) HIP HOP sensitivity profile for 4′‐hydroxychalcone at the IC_50_ concentration (97 μm).

In order to validate the simplified HIP HOP panel, we tested it with the known antifungal drug caspofungin which is a well characterised inhibitor of chitin cell wall biosynthesis. Using Table 1, we can see that various cell wall synthesis inhibitors have previously been screened using genome wide HIP HOP profiling and that certain genes involved with cell wall synthesis are diagnostic for this mechanism of action. From the information presented in Table 1, the HIP strains *rho1*Δ/*RHO1* and *fks1*Δ/*FKS1* are predicted to be diagnostically hypersensitive to cell wall synthesis inhibitors. To show that our simplified HIP HOP assay is able to correctly identity a cell wall synthesis inhibitor, we then tested caspofungin at 0.15 μg·mL^−1^ which corresponds to half the minimal inhibitory concentration observed with the BY4743 parental yeast strain. The results (Fig. 1B) show a heatmap representing inhibition relative to untreated controls of each yeast deletion strain. Figure 1B shows that as expected the strains *rho1*Δ/*RHO1* and *fks1*Δ/*FKS1* are indeed the most sensitive of all strains in the HIP panel. Furthermore, from the HOP panel, Table 1. suggests the strains *chs3*Δ/*chs3*Δ, *skt5*Δ/*skt5*Δ and *chs6*Δ/*chs6*Δ as diagnostic for inhibition of cell wall synthesis and as expected they are among the most sensitive in the HOP panel. Also, among the most sensitive HOP strains is *npl3*Δ/*npl3*Δ which is not a specific cell wall synthesis gene. This strain was also reported as amongst the most sensitive HOP strains in the original Novartis screen, therefore it is not surprising it would appear as hypersensitive in our simplified HIP HOP assay. Therefore, the results with caspofungin demonstrate the importance of looking for groups of genes with related functions that act to corroborate a particular mode of action.

### The profile of trans‐chalcone and 4′‐hydroxychalcone suggests a promiscuous mode of action but with transcription or translation featuring prominently

**Fig. 2:**
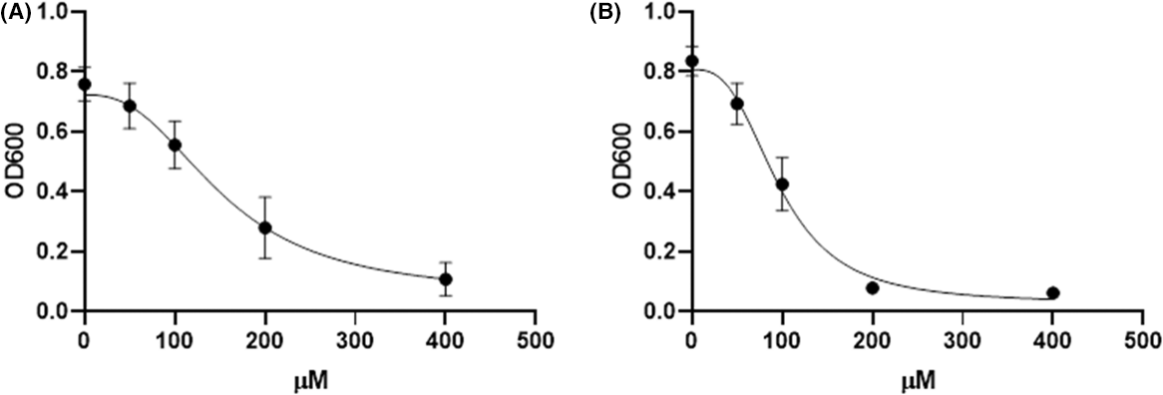
Dose–response growth inhibition of BY4743 yeast with (A) *trans*‐chalcone (IC_50_ 153.5 μm) and (B) 4′‐hydroxychalcone (IC_50_ 97 μm). Error bars indicate SEM *n* = 8.

Next, we tested two yeast inhibitory chalcone compounds, *trans*‐chalcone and 4′‐hydroxychalcone. Despite their importance in representing the basic chalcone pharmacophore, neither compound is well characterised in terms of their mechanism of action. Dose–response inhibition curves are shown in Fig. 2, *trans*‐chalcone (A) is the less active of the two compounds with an IC_50_ of 153.5 μm compared to 97 μm for 4′‐hydroxychalcone (B).

**Fig. 3:**
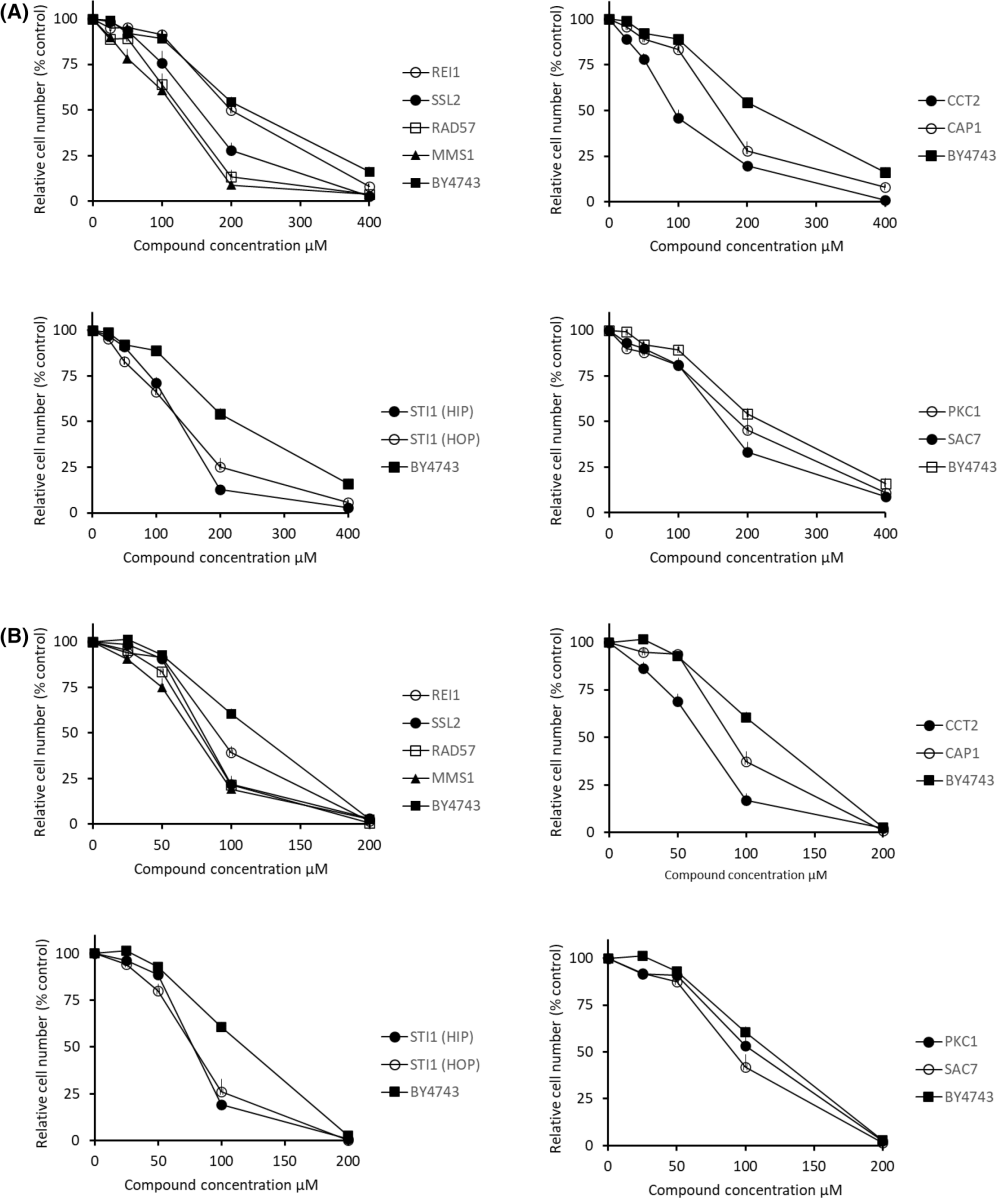
Dose–response inhibition of growth by (A) *trans*‐chalcone and (B) 4′‐hydroxychalcone using selected hypersensitive strains from the HIP HOP screen. Error bars indicate SEM *n* = 5.

Having established a yeast growth inhibition IC_50_ value for each compound, the yeast panel was then screened at the corresponding IC_50_ with each compound (Fig. 1C,D). Further validation of the heatmap hypersensitivity profile was obtained by retesting a selection of the top 10% most hypersensitive strains using dose–response growth inhibition experiments (Fig. 3A,B). Preference for testing was given to strains that presented as hypersensitive with a corresponding HIP or HOP strain that corroborated the mechanism. Rather than revealing a single mechanism of action, the HIP HOP profile shows perturbation of a range of different cellular processes thereby revealing that both compounds act in a highly promiscuous manner. The genes involved represent a range of separate areas of cell biology, namely DNA‐associated processes, the actin cytoskeleton, the chaperone HSP90 and protein kinase C. Interestingly, both profiles show similar patterns of hypersensitivity among the gene deletion strains represented in the panel, as might be expected for two highly related compounds, thus indicating that although promiscuous, both compounds act through similar mechanisms.

Overall, both profiles hint at inhibition of transcription, translation or DNA damage as possible mechanisms of action. This can be seen in the HIP panel with hypersensitivity observed in *rei1*Δ/*REI1*, *nmd3*Δ/*NMD3* and *ssl2*Δ/*SSL2* each of which are diagnostic for DNA intercalators that are known to induce transcriptional stress. For both compounds, this characteristic grouping is further supported in the HOP profile by the DNA repair deletion strains *rad54*Δ/*rad54*Δ, *rad55*Δ/*rad55*Δ, *rad57*Δ/*rad57*Δ and *mms1*Δ/*mms1*Δ. Possible explanations for this grouping include, DNA binding, inhibition of translation/transcription or induction of DNA damage *via* inhibition of topoisomerase II.

**Fig. 4:**
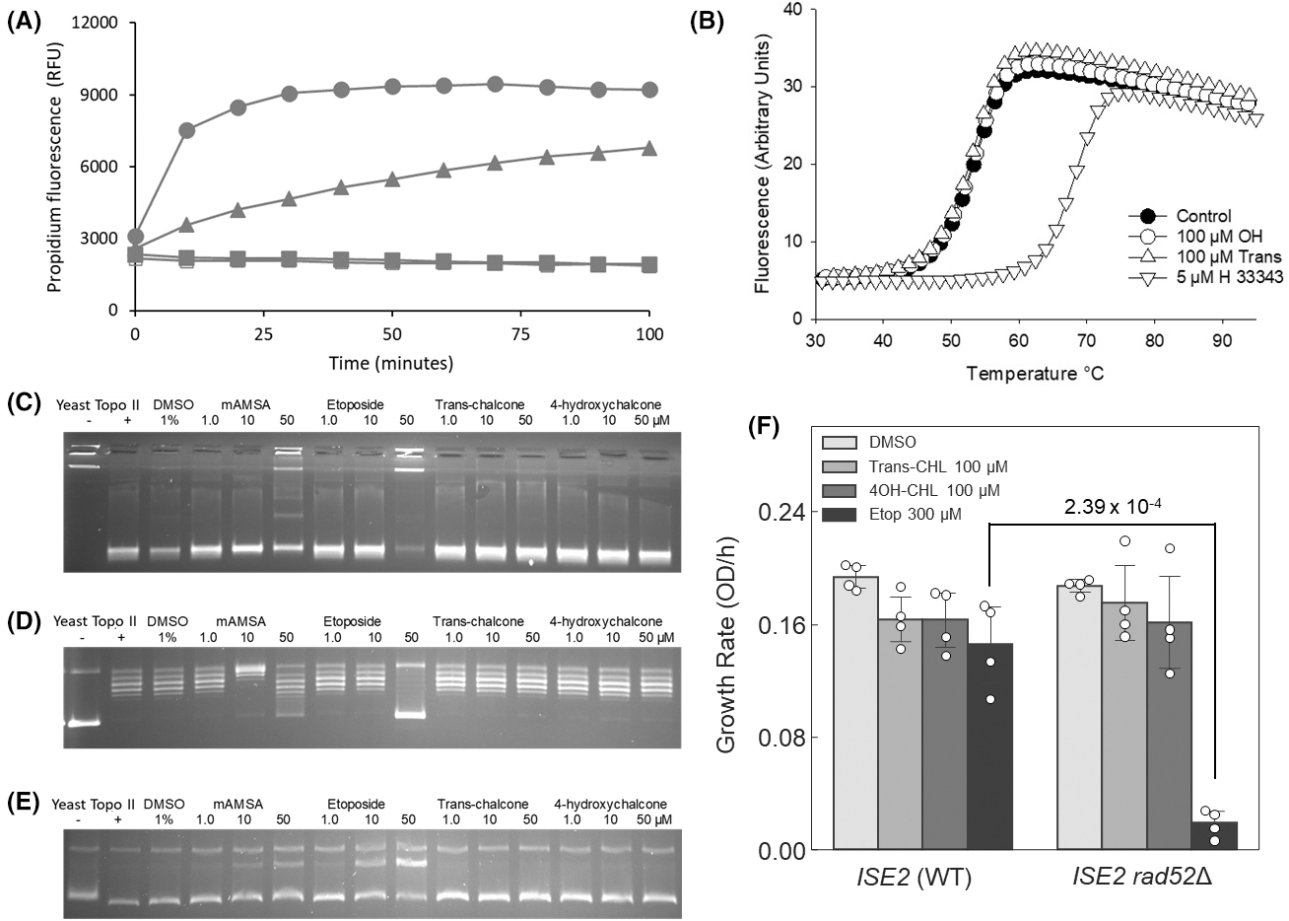
(A) Neither chalcone compound induces membrane damage that is detectable using propidium iodide, whereas SDS induces membrane damage as expected. One hundred micromolar SDS (circles), 50 μm SDS (triangles), trans‐chalcone (filled squares) and 4′‐hydroxychalcone (open squares, overlapping with filled squares). (B) DNA binding is eliminated as mechanisms of action. DNA melting curves in the presence of 100 μm 4′‐hydroxychalcone (open circles), 100 μm *trans*‐chalcone (open triangles) reveal neither compound to be a DNA intercalator. Five micromolar of Hoechst 33343 (inverted triangles) are included as a positive control along with a no treatment control (filled circles). (C) Assay of *trans*‐chalcone and 4′‐hydroxychalcone with yeast topoisomerase II in a decatenation assay. (D) Assay of *trans*‐chalcone and 4′‐hydroxychalcone with yeast topoisomerase II in a relaxation assay. (E) Assay of *trans*‐chalcone and 4′‐hydroxychalcone with yeast topoisomerase II in and a DNA cleavage assay. In each case the compounds do not inhibit topoisomerase II. In each panel, lane 1 is a native DNA control substrate and lane 2 is a control of decatenated DNA, relaxed DNA or supercoiled DNA respectively. The DNA intercalator mAMSA and the control topoisomerase II poison etoposide are included as positive controls. (F) In support of this, neither compound exhibits hypersensitivity with a *rad52* deletion strain. Both chalones were tested and compared to an equipotent concentration of the control topoisomerase II poison etoposide. Growth rates were obtained from real‐time growth curves (mean ± SD, *n* = 4). Statistical significance was calculated with a two‐sided unpaired *t* test.

The heatmap also eliminates certain mechanisms of action. For example, none of the membrane damage set of diagnostic strains exhibit hypersensitivity in the HIP profile with either compound (see *neo1*Δ/*NEO1*, *pik1*Δ/*PIK1*, *tim54*Δ/*TIM54*, *cmd1*Δ/*CMD1*, *tda10*Δ/*TDA10*) and in the HOP panel the same is true for *lem3*Δ/*lem3*Δ. Thus, neither chalcone compound exhibits the pattern of hypersensitivity reported in Table 1 for compounds known to have a membrane damaging off‐target mechanism, for example amitriptyline and chlorpromazine [21, 22]. As independent confirmation of this, both compounds were then tested for perturbation of the plasma membrane using propidium iodide which exhibits increased fluorescence when bound to DNA if able to enter the cell as a result of membrane damage. As expected (Fig. 4A) the known membrane disrupting surfactant sodium dodecyl sulphate (SDS) induced an increase in propidium fluorescence caused by propidium entering the cells as a result of membrane damage, whereas neither chalcone was able to create the same effect, even at 100 μm.

In addition to membrane damage, a range of other mechanisms are rendered unlikely. For example, the enzyme topoisomerase 1 has been suggested as a molecular target for certain chalcones which are able to exert DNA damage *via* poisoning of topoisomerase 1. In this mechanism it is recruitment of topoisomerase 1 into a DNA damaging enzyme that results in growth inhibition as opposed to catalytic inhibition of topoisomerase enzyme activity. For this reason, yeast completely lacking a functional copy of topoisomerase 1 such as the homozygous deletion strain *top1*Δ/*top1*Δ in the HOP panel is diagnostically resistant to topoisomerase 1 poisons such as camptothecin [4, 23]. Figure 1 shows that the *top1*Δ/*top1*Δ strain is not resistant to either chalcone compound thereby eliminating this as a possible mechanism. Indeed, as *TOP1* is non‐essential and is completely deleted in the HOP strain, the continued activity of both compounds in this strain is strong evidence against topoisomerase 1 poisoning as a key mechanism in yeast.

### DNA binding and topoisomerase II inhibition are eliminated as potential mechanisms

One potential mechanism of action suggested by the HIP HOP profile is that both chalcones are DNA binders. For example, known DNA intercalators such as quinacrine, Hoechst 33258 and actinomycin D provide a similar profile with hypersensitivity seen in HIP strains *rei1*Δ/*REI1*, *nmd3*Δ/*NMD3* and *ssl2*Δ/*SSL2*. Indeed, DNA binding has been suggested as a mechanism of action for a range of related chalcone compounds [18]. To test this potential mechanism, both chalcone compounds were assayed for DNA binding using duplex DNA oligomers labelled with the fluorophore (fluorescein) at the 5′‐end of one DNA strand (5′‐F‐AAAAAAACGTGAAAAAA), while a quencher (dabcyl) was attached to the 3′‐end of the complementary strand (3′‐Q‐TTTTTTTGCACTTTTTT). This provides a sensitive assay for DNA binding by small molecules which would be expected to change the melting temperature of the DNA duplex and result in a shift in the melting curve which can be detected by fluorescence. The results (Fig. 4B) show that while the known DNA binder Hoechst 33343 causes a characteristic shift in melting temperature, neither of the two chalcone compounds change the melting temperature even at 100 μm thereby eliminating DNA binding as a mechanism to explain the growth inhibitory effects of the two compounds. Furthermore, we tested for any evidence of intercalation using a plasmid‐based intercalation assay and found no evidence for intercalation by either compound (data not shown).

Another potential mechanism to explain the HIP HOP profile is topoisomerase II poisoning, which has been reported as a mechanism of action for related chalcone compounds [17]. In this mechanism, so called poisoning of topoisomerase II results in toxic DNA double strand breaks. To test this possibility, both chalcone compounds were assayed with yeast topoisomerase II in decatenation, relaxation and cleavage assays. The results (Fig. 4C–E) show that unlike the positive control DNA intercalator mAMSA and the known topoisomerase II poison etoposide, neither *trans*‐chalcone or 4′‐hydroxychalcone inhibit topoisomerase II decatenation, relaxation or induced DNA cleavage. An alternative possibility is that although neither compound poisons topoisomerase II directly, biotransformation by yeast results in topoisomerase poisoning reaction products. In order to address this, we compared both chalcones against equipotent concentrations of etoposide in a strain that is both sensitive to etoposide and also deficient in the repair of the DNA damage caused by topoisomerase II poisons (*ISE2 rad52*Δ) [24, 25]. Whereas etoposide induced hypersensitivity in *ISE2 rad52*Δ relative to the isogenic control *ISE2 RAD52*, the same effect was not seen with either chalcone compound (Fig. 4F). Thus, topoisomerase II poisoning or inhibition of topoisomerase catalytic activity is not a mechanism that is able to explain the biological activity of the two chalcones.

### Chalcone activity is associated with oxidative stress but the effects are sub‐lethal

**Fig. 5:**
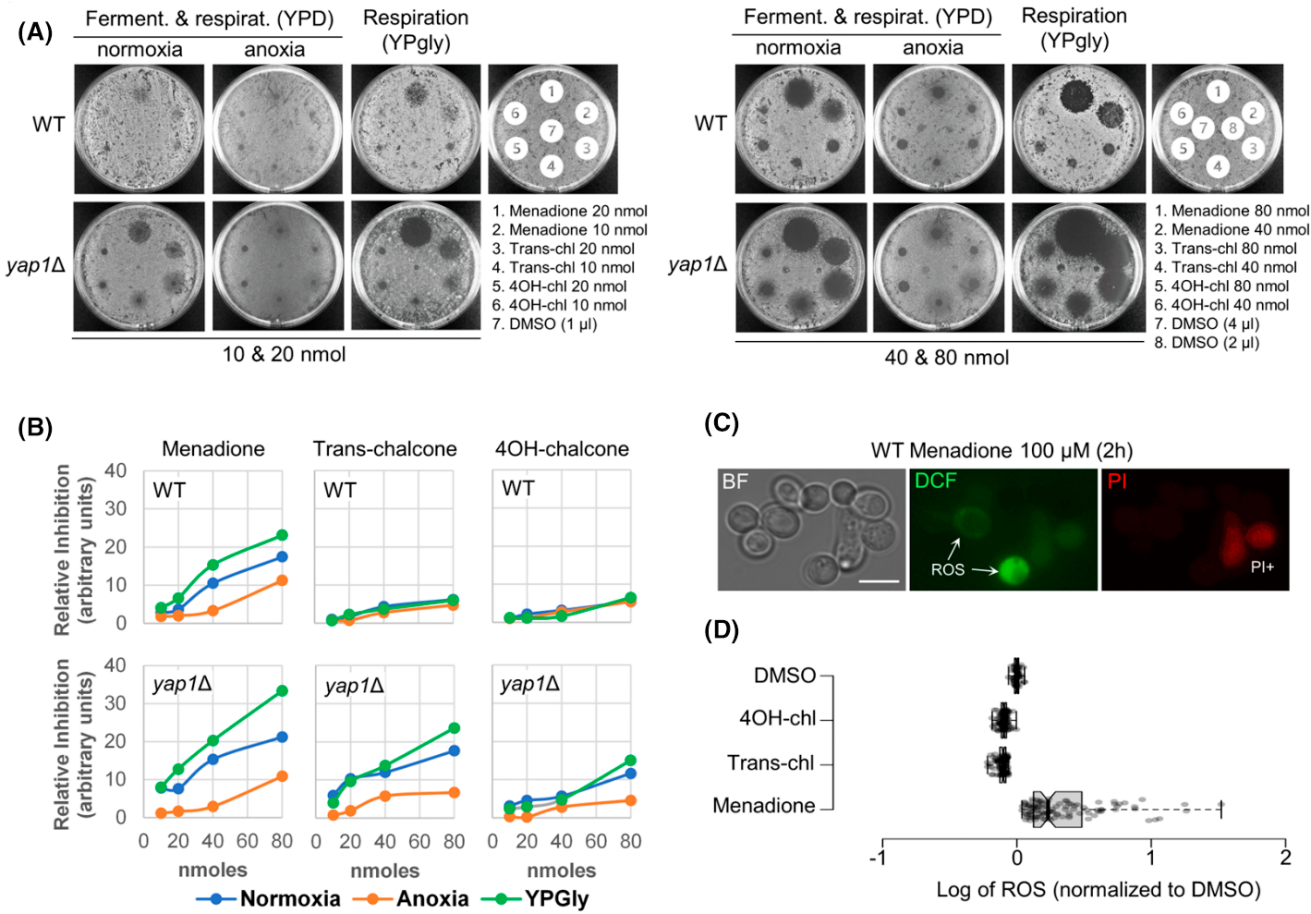
Contribution of oxidative stress to chalcone toxicity. (A) Halo of growth inhibition for BY4741 and *yap1*Δ in fermentable conditions (glucose) and under normoxia and anoxia (48 and 72 h, respectively). Additionally, normoxic inhibition halos for the same strains under strict respiration conditions (glycerol, 72 h) are shown. Four equimolar amounts of compounds were used: 10 and 20 nmoles (left), as well as 40 and 80 nmoles (right). (B) Quantification of growth inhibition based on the halo profiles. (C) Representative micrographs of ROS generation after menadione addition as determined with DCFH‐DA (non‐fluorescent) or DCF (green fluorescent) conversion. PI+ cells are excluded in DCF measurements. Scale bar corresponds to 5 μm. BF, bright field. (D) Quantification of ROS (DCF mean intensities per cell) 2 h after addition of the corresponding compound (100 μm).

The HOP profile revealed hypersensitivity in *yap1*Δ/*yap1*Δ with both chalcones. As oxidative stress has been proposed as a mechanism of action for chalcones in human cells [26], we investigated whether oxidative stress plays a role in the cell inhibitory effects of the two chalcone compounds in yeast. Yap1 is a master regulator in the oxidative stress response [27]. As *S. cerevisiae* can be grown in the complete absence of oxygen, we reasoned that if *yap1*Δ hypersensitivity to chalcones was related to oxidative stress, both compounds should show increased activity in yeast incubated in the presence of oxygen (normoxia) compared to yeast incubated in the absence of oxygen (anoxia). We included menadione as a positive control, as it is a well‐studied quinone whose toxicity depends on oxidative stress [28]. As expected, the *yap1*Δ strain was hypersensitive to menadione, and anoxic conditions greatly diminished menadione yeast inhibition in both the wild type and *yap1*Δ (Fig. 5A,B). Likewise, the inhibition halo was larger in the *yap1*Δ strain growing in glycerol, which supports ROS generation [29]. The halo profiles of both chalcones mimicked those of menadione in the *yap1*Δ strain. However, in wild‐type yeast that is not deleted for *YAP1*, the same pattern was not observed, and the presence or absence of oxygen did not affect chalcone inhibitory activity (Fig. 5A,B). This indicates that although ROS generation is occurring it likely exists as a sub‐lethal mechanism. In other words, Yap1‐deficient yeast that lack a viable defence to oxidative stress are hypersensitive to both chalcones, but this effect does not occur in wild‐type yeast that are Yap1 proficient and is therefore not a key mechanism. To confirm this, we measured ROS in wild‐type yeast in the presence of the two chalcones and compared ROS levels to those obtained with menadione and DMSO, however ROS levels after chalcone addition did not differ from DMSO (Fig. 5C,D).

### Both chalcone compounds inhibit transcription *in vivo*

**Fig. 6:**
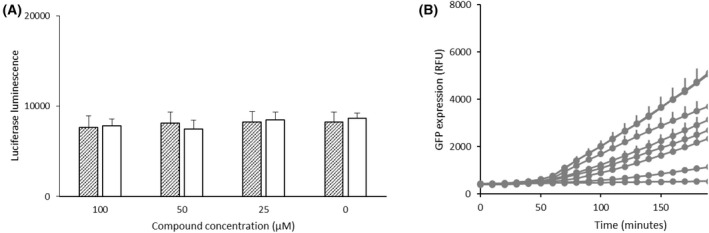
(A) Neither *trans*‐chalcone (hatched bars) or 4′‐hydroxychalcone (open bars) inhibit translation in a cell‐free system. Both compounds were incubated at specified concentrations with rabbit reticulocyte lysate, amino acids and luciferase mRNA. Translation of mRNA into functional luciferase was measured by luminescence. Error bars indicate SEM *n* = 3. (B) GFP expression from an inducible GAL promoter in response to different inhibitor compounds. Bottom line (G) is no galactose induction control, all other lines are with galactose induction of GFP. Two hundred micromolar of 4′‐hydroxychalcone (F), 100 μm 4′‐hydroxychalcone (E), 200 μm *trans*‐chalcone (D), 100 μm *trans*‐chalcone (C), 50 μm actinomycin D (B). The top two lines which overlap to appear as single line (A) are DMSO only or 50 μm fluconazole. Error bars are SEM *n* = 8.

Although the HIP HOP profile demonstrates highly promiscuous mechanisms are at play (rather than a single targeted mechanism), nonetheless many of the more hypersensitive responses are associated with genes involved in transcription, translation and DNA repair. For example, in the HIP profile *rps16b*Δ/*RPS16B*, *rpl31a*Δ/*RPL31A* as well as *iml2*Δ/*IML2* (ribosomal proteins) appear hypersensitive and in the HOP profile so do *rps24a*Δ/*rps24a*Δ and *rps10b*Δ/*rps10b*Δ (also ribosomal proteins). Furthermore, in the HIP profile *rei1*Δ/*REI1*, *nmd3*Δ/*NMD3* and *ssl2*Δ/*SSL2* appear hypersensitive, an attribute shared with quinacrine and the RNA polymerase inhibitor actinomycin D, each of which induce transcriptional stress (Table 1). As transcription and translation are closely intertwined, compounds that inhibit transcription can induce a hypersensitive response in strains deleted for ribosomal proteins. For example, the HIP HOP profiles of quinacrine and actinomycin D, are both enriched for ribosomal proteins [4]. To test the possibility that both chalcones act through a translation inhibiting mechanism, both compounds were tested for their ability to inhibit cell‐free translation using rabbit reticulocyte lysate. This assay includes all the functional components needed to recapitulate translation *in vitro* and makes use of a luciferase‐encoding mRNA to allow expression of luciferase, which can then be detected using luminometry. The results (Fig. 6A) show that neither compound inhibits translation even at 100 μm concentrations, thereby eliminating translation as a potential mechanism of action.

Having eliminated translation, DNA binding and topoisomerase II poisoning, a remaining possibility suggested by the HIP HOP profile is induction of transcriptional stress. A generalised transcriptional stress mechanism would explain the high number of DNA‐associated genes in the hypersensitive regions of the HIP HOP profiles for both compounds. To test this possibility, we made use of inducible expression of GFP in a yeast strain using an inducible *GAL1* promoter, to see if either chalcone could rapidly act to inhibit transcription *in vivo*. The known inhibitor of transcription, actinomycin D, was included as a positive control. An additional control of fluconazole was also included. Fluconazole is highly inhibitory to yeast *via* inhibition of ergosterol synthesis but does not act directly on transcription or translation and is therefore a useful negative control compound. Figure 6B shows that GFP expression is induced or not induced by the presence or absence of galactose as expected (lines A and G). Additionally, actinomycin D, the known inhibitor of transcription, inhibits inhibition of GFP expression as expected (line B), whereas fluconazole which is not a direct inhibitor of transcription or translation does not induce inhibition as expected (line A overlapping with DMSO vehicle control). For the chalcones, interestingly both compounds inhibit GFP expression and in the same order by which they inhibit the growth of *S. cerevisiae*. For example, 4′‐hydroxychalcone is the more active of the two compounds in terms of yeast growth inhibition and is also the more active inhibitor of GFP‐expression. For *trans*‐chalcone, inhibition of GFP expression increases between 100 and 200 μm (lines C and D). By comparison, 4′‐hydroxychalcone is more active; inhibition of GFP expression increases between 100 and 200 μm (lines E and F) with 200 μm (F) reaching near total inhibition.

## Discussion

Determining the mechanism of action of a novel compound is a complex process and is fraught with difficulties. Recent problems reported in reproducing published mechanisms of action of lead cancer compounds suggest off‐target mechanisms may play a role in unexpected toxicity [7, 8] and it is likely the same principle applies to antifungal drug discovery. Yeast chemogenomic profiling provides a useful solution to identifying mechanisms of action for a novel compound, and if used early on in an investigation can identify off‐target toxicity, ensuring compounds do not unnecessarily enter animal studies. Due to conservation of fundamental biological processes between yeast and humans, such assays can provide mechanistic insights for novel small molecules targeting cancer‐related processes, but they are also highly valuable tools in antifungal drug discovery [4]. In this study, we have demonstrated how a simplified yeast chemogenomic profiling method can be used to gain insights into the cell inhibitory mechanism of action of two related chalcone compounds. The assay is extremely simple to perform, requires minimal test compound and is highly cost effective. As the yeast strains are readily available from Euroscarf, this highly simplified screening tool provides an accessible approach for natural product researchers to examine the mechanism of action of antifungal or cytotoxic compounds. We applied the assay to two chalcone compounds. The resulting chemogenomic profiles suggest the compounds are promiscuous and affect numerous different areas of cell biology but with transcriptional stress playing a role in their biological effects. Future studies examining chalcone mechanisms of action of should examine transcriptional stress as a potential off‐target mechanism before initiating more detailed investigations of targeted ligand‐receptor type mechanisms.

## Author contributions

TAKP conceived of project, wrote the first draft of the manuscript, with contributions to subsequent drafts made by LA‐A, KRF, AM, BP and FM. TAKP carried out selection of gene deletion strains, performed the HIP HOP screen and transcription/translation assays; FM carried out DNA damage and oxidative stress assays as well as ROS determination; LA‐A carried out DNA damage and oxidative stress assays; KRF carried out DNA melting curve assays; AM carried out topoisomerase assays; BP made the GFP reporter strain.

## Data Availability

Data available on request from the authors.
